# Correction: The Effect of Paternal Age on Offspring Intelligence and Personality when Controlling for Paternal Trait Level

**DOI:** 10.1371/journal.pone.0097370

**Published:** 2014-05-05

**Authors:** The PLOS ONE Staff

## Notice of Republication

This article was republished on April 21, 2014 to correct errors in the title. The correct title is “The Effect of Paternal Age on Offspring Intelligence and Personality when Controlling for Parental Trait Levels.” Please download this article again to view the correct version. The originally published, uncorrected article and the republished, corrected article are provided here for reference.

## Supporting Information

File S1 Originally published, uncorrected article.
(PDF)

File S2 Republished corrected article.
(PDF)
