# Supplementary material for: Correction: The Effect of Paternal Age on Offspring Intelligence and Personality when Controlling for Paternal Trait Level
Source: PLoS One. 2014 May 5;9(5):e97370. doi: 10.1371/journal.pone.0097370 (PMC4010487; doi:10.1371/journal.pone.0097370)
Supplement: File S1 — Originally published, uncorrected article. (PDF) [file pone.0097370.s001.pdf]

OPEN ACCESS Freely available online

PLOS | ONE

The Effect of Paternal Age on Offspring Intelligence and Personality when Controlling for Paternal Trait Level

Ruben C. Arslan[1,2]*, Lars Penke[2], Wendy Johnson[3], William G. Iacono[4], Matt McGue[4]

1 Institute of Psychology, Humboldt University of Berlin, Berlin, Germany, **2** Institute of Psychology, Georg August University Göttingen, Göttingen, Germany, **3** Department of Psychology and Centre for Cognitive Ageing and Cognitive Epidemiology, University of Edinburgh, Edinburgh, United Kingdom, **4** Department of Psychology, University of Minnesota, Minneapolis, Minnesota, United States of America

Abstract

Paternal age at conception has been found to predict the number of new genetic mutations. We examined the effect of father's age at birth on offspring intelligence, head circumference and personality traits. Using the Minnesota Twin Family Study sample we tested paternal age effects while controlling for parents' trait levels measured with the same precision as offspring's. From evolutionary genetic considerations we predicted a negative effect of paternal age on offspring intelligence, but not on other traits. Controlling for parental intelligence (IQ) had the effect of turning an initially positive association non-significantly negative. We found paternal age effects on offspring IQ and Multidimensional Personality Questionnaire Absorption, but they were not robustly significant, nor replicable with additional covariates. No other noteworthy effects were found. Parents' intelligence and personality correlated with their ages at twin birth, which may have obscured a small negative effect of advanced paternal age (<1% of variance explained) on intelligence. We discuss future avenues for studies of paternal age effects and suggest that stronger research designs are needed to rule out confounding factors involving birth order and the Flynn effect.

Citation: Arslan RC, Penke L, Johnson W, Iacono WG, McGue M (2014) The Effect of Paternal Age on Offspring Intelligence and Personality when Controlling for Paternal Trait Level. PLoS ONE 9(2): e90097. doi:10.1371/journal.pone.0090097

Editor: Antonio Verdejo García, University of Granada, Spain

Received July 24, 2013; **Accepted** January 28, 2014; **Published** February 25, 2014

Copyright: © 2014 Arslan et al. This is an open-access article distributed under the terms of the Creative Commons Attribution License, which permits unrestricted use, distribution, and reproduction in any medium, provided the original author and source are credited.

Funding: The authors have no support or funding to report.

Competing Interests: The authors have declared that no competing interests exist.

* E-mail: ruben.arslan@gmail.com

Introduction

The well-established genetic influences on psychological traits such as intelligence and personality traits have attracted the attention of a growing number of evolutionary psychologists. This is because selection continues to exert pressure on heritable traits, unless they are completely irrelevant for fitness. There is evidence that neither intelligence nor personality traits are currently completely neutral to selection, but are associated with fitness components like survival [1–3] and reproductive outcomes [4–7]. If we make the assumption that at least somewhat similar selection processes affected these traits with some consistency during the last few thousand years we must wonder why genetic differences in these traits persist [8]. They imply the existence of maintaining evolutionary mechanisms, since otherwise natural selection would drive variants to fixation and eliminate the differences [8,9].

Here we tested the hypothesis that harmful genetic mutations that occur anew each generation might contribute to the genetic variation particularly of intelligence, which would suggest that this genetic variation is maintained by a balance of mutation and counteracting selection. To test this hypothesis, we relied on paternal age at twin birth (henceforth simply "paternal age") as a proxy of new mutations and used a better-controlled design than previous studies. We review the increasingly supportive evidence for paternal age as an indicator of new mutations as well as the importance of using the right controls after explaining the evolutionary genetic reasoning behind the hypothesis that mutations contribute substantially to the genetic variation in intelligence.

1. Evolutionary Explanations for Individual Differences

Because intelligence is regarded as an attractive trait in mates across cultures [10–13], it is plausible that higher intelligence was also preferred during recent human evolutionary history. Thus, high intelligence could be positively sexually selected, driving low intelligence to extinction (barring evolutionarily very novel impediments like effective birth control; [14,15]). There is also evidence for survival selection for intelligence in current times [3], though it is of course hard to infer how the relation between intelligence and survival has varied during evolutionary history. To explain why high intelligence has not been fixated, Penke et al. [9] argued that intelligence has a large number of relevant genetic loci and thus presents a large target for mutations. Mutational target size includes loci that are not polymorphic, but whose alteration would affect the trait. It thus includes a larger number of loci than those which are currently polymorphic and might be picked up by analyses of common single nucleotide polymorphisms (SNPs). As such a target, intelligence would be under *mutation-selection balance*, which occurs when purifying selection removes mutations deleterious to fitness from the gene pool, but cannot outpace the occurrence of new mutations. Thus, a number of mutations persist in the population and individuals have varying "mutation loads" [16]. The predicted effect of genetic perturbations depends on whether the trait is under stabilising or directional selection. Stabilising selection leads to a buffering

against both deleterious and beneficial changes (robustness), whereas under directional selection leads to a higher evolvability or responsiveness to perturbations [17]. Higher robustness would imply smaller effects of new mutations [18]. Brain size was previously held to be a good evolutionary proxy of intelligence [19]. The brain, as a bone-encased organ, however, may have been under predominantly stabilising selection due to anatomical and developmental constraints, while intelligence is still often thought to have been under directional selection [20]. Therefore, we predict that indicators of mutation load should be negatively related to intelligence, but not so much to brain size.

Penke et al. [9] contrasted personality traits with intelligence to show that they are not just distinct due to convention or methods, but a product of different selection pressures. They argued that personality variation does not fit the pattern evoked by mutation-selection balance and predicted only a medium mutational target size for personality variation. They suggested that this favoured *balancing selection* as the explanation for personality differences. Balancing selection is a class of mechanisms in which fitness effects of a trait variant differ by environment, be it spatial, social, temporal or genetic (i.e. epistasis and overdominance) [21]. Variation is thus maintained by selection of different trait levels in different environments.

Recent genome-wide complex trait analyses found that more of the genetic variation of intelligence [22–25] than of personality traits [26,27] is associated with small genetic relationships captured by common genetic variants that have high frequencies in the population and are thus unlikely to be novel harmful mutations. Despite this, some of the genetic variation remains unexplained in these traits. Furthermore, the weak signals from the common genetic markers in these studies might come from older mutations in linkage disequilibrium with the markers, or even from novel rare variants in weak linkage disequilibrium but with strong effects on the phenotype [28–32]. In fact, we know of more than 300 of rare mutations that have major effects on intellectual ability [33–35]. Potential participants with intellectual disability are usually excluded from research on intelligence in the normal range. Still, substantial evidence points toward mutation-selection balance [36] as a reasonable explanation for much of the genetic influence on intelligence. However, these and other molecular genetic findings [36–38] cast doubt on balancing selection as the main explanation for genetic personality variance. Instead, a more differentiated view of different personality domains is spreading [26,39–41]. Still, in the absence of a new convincing pattern implicating a specific mechanism in personality, balancing selection may still be a viable explanation. Therefore we predict that indicators of mutation load are unrelated to personality traits.

2. Genetic Mutations and Paternal Age

Mutation-selection balance can occur because mutations are generally much more likely to harm the intricate system they affect than to add adaptive benefits to it [42], so the expected effect of new mutations is in the opposite direction of selection. But where do new mutations originate? To maintain mutation-selection balance, mutations need to be inherited, so they need to be germline, not somatic, mutations. Keightley's [42] estimates were in line with Kong et al. [43], who reported an average of 63.2 new mutations when comparing the sequenced whole genomes of parent-offspring trios. Keightley [42] also estimated that on average 2.2 of these new mutations per generation are deleterious (reducing fitness), which would be implausibly high if each mutation had to be eliminated through failure of the carrier to reproduce, but not if selection acts on relative fitness differences among individuals (quasi-truncation selection [44,45]).

Keightley [42] reviewed the available evidence and found that most mutations are paternal in origin, as had been suggested for a long time [46–48]. His finding was corroborated by Campbell et al. [49] and Kong et al. [43]. The latter reported 3.9 times higher mean single nucleotide mutations of paternal than maternal origin. Strikingly, the far-larger heterogeneity (ratio of variances = 8.8) in male mutation rates could almost entirely be accounted for by paternal age; Kong et al. [43] reported an estimated increase in paternal mutations with age of two per year. Crow [50] found single nucleotide mutations in which one nucleotide had been mis-transcribed into one of the other three to be far more common during male than female gametogenesis. Originally, the suspected reason for this was the far greater number of pre-meiotic cell divisions in sperm (35+23 * years after puberty) compared to oocytes (24) leading to an accumulation of errors with age. New data is consistent with this linear relationship, but there is also evidence for "selfish spermatogonial selection" (i.e. pre-meiotic selection for mutated cells) at a few loci [51–55]. Decay of transcription fidelity, proofreading error, or some combination of these pathways [50,56] may also be involved.

Single nucleotide mutations appear to be the most common type [57], though they do not account for the most altered base pairs per birth [51]. Unlike chromosomal aberrations, which affect the most base pairs but are unlikely to explain normal variation in the traits considered here, such as trisomy 16 and 21, they do not occur more often with advancing maternal age [42,43,58]. Like single nucleotide mutations, new copy number variants (CNV; duplicated or deleted base pair sequences) also seem to have a paternal origin bias [51] and to be associated with increasing paternal age in mouse models [59]. Molecular genetic analyses in humans seem to yield different biases for different types of CNVs, with a paternal age bias having been found for CNVs with non-recurrent breakpoints, but not for others [51,60].

Because epigenetic insults accumulate in somatic cells during a lifetime [61], there has been speculation that paternal age effects could potentially be explained through epimutations [62,63], though erasure of epigenetic information in the germline is thought to limit if not prevent their inheritance [61].

To summarize, since paternal age at conception is linearly related to the number of pre-meiotic cell divisions, it can be used as a proxy for likelihood of new germline mutations [43].

3. Paternal Age and Psychological Traits

Keller and Miller [64] and Uher [65] argued that severe mental illnesses that confer strong reproductive disadvantages should owe their continued existence to pleiotropic effects of rare recent mutations. Indeed, the increased likelihood of schizophrenia in offspring of older fathers is well documented [66] and has been noted since the 1950s [67] and more recently by Malaspina et al. [68]. Reichenberg et al. [69] reported similar observations for autism, as did Frans et al. [70] for bipolar affective disorder and Lopez-Castroman et al. [71] for intellectual disability. By contrast, effects seem to be trivial or zero for unipolar depression [72]. Paternal age associations with sporadic (nonfamilial) cases of Apert's syndrome, achondroplasia, progeria and other diseases have been found consistently [65]. For autism, schizophrenia and intellectual disability, paternal age effects have recently been corroborated by exome-sequencing studies [35,73–75], some of which also reported auxiliary analyses of the association of paternal age with IQ.

Searching for a stable phenotype associated with schizophrenia led Malaspina et al. [76] to examine the relation of paternal age with IQ in the general population. In a large ($N = 44{,}175$) sample of Israeli conscripts, they reported a shallow inverted U-shaped

relation with IQ, which is a risk factor for schizophrenia [77]. The relation persisted even after controlling for maternal age, parental education and numerous other possible confounds. One subsequent, independent study replicated the finding in a large ($N = 33{,}437$) sample of children for several intelligence measures across three waves [78]. However, after controlling for maternal education, birth order, birth weight and family size in the same sample, Edwards and Roff [79] found many of the associations reduced to non-significance. They argued for the added controls, but Svensson, Abel, Dalman, and Magnusson [80] expressed concern that the correction for birth weight might remove a mediated effect [81,82] and make a real association look spurious. Still, the largest effect reduction resulted from controlling for maternal education. This choice can hardly be contested, because maternal education can be a proxy for heritable maternal intelligence and Edwards and Roff [79] showed that maternal education correlated negatively with father's age due to period effects on education in their cross-sectional sample. Svensson et al. [80] did not find a negative link between paternal age and scholastic achievement in adolescence either. Their sample (the largest so far; $N = 155{,}875$) comprised recent birth cohorts in Stockholm county, where delayed paternity is common. They controlled maternal and paternal education (not scholastic achievement), country of birth, parental mental health service use and graduation year (to control rising grades).

Auroux et al. [83], who had previously reported a negative association between advanced paternal age and military aptitude test scores [84], did not replicate that relation in newer data ($N = 6{,}564$) when controlling for parents' academic level, but instead found an association between lower paternal age and lower aptitude. Similarly, Whitley et al. [85] found lower IQs for children born to younger fathers after controlling for number of older siblings ($N = 772$). However, in the same sample they found no association between paternal age and reaction time, arguably a measure less influenced by cultural, social and educational background.

The same research groups who found negative links between paternal age and intelligence also reported associations in the same general population samples between paternal age and aspects of personality, namely poor social functioning [86] and externalising behaviour [87]. Lundström et al. [88] reported a U-shaped relation of paternal age with autistic-like normal variation in two Swedish twin samples, though Robinson, Munir, McCormick, Koenen, and Santangelo [89] could not replicate this finding in a smaller sample.

An explanation of the paternal age effects that relies on new mutations or epigenetics [63] mandates thorough control for alternative explanations. An obvious possibility is that parental personality [90] and intelligence [15] influence reproductive timing and therefore paternal age. Offspring's inherited personality and intelligence would then differ according to paternal age because of this unobserved common cause. So far, parental intelligence and personality as confounds have not been ruled out, because only proxy variables like education or socioeconomic status (SES) were available in the samples. Proxies for personality measures have not yet been controlled in any study, to our knowledge.

The effect of controlling for familial predisposition has been studied more when it comes to mental illnesses by comparing familial and sporadic cases. With continuously measured traits like intelligence and personality, we can hope to control for the parental contribution with greater precision.

Statistical controls for parental traits are still necessary when new mutations are directly quantified. However, in three recent clinical exome-sequencing studies, such controls were not possible and the reported associations with intelligence may thus have been biased: Iossifov et al., [91] and Sanders et al. (2012) [58] counted new SNPs by comparing parents' and children's exomes. They reported no links between new rare SNPs and intelligence. Sanders, et al. (2011) [92], on the other hand, reported a negative association with CNVs. Generalizability may be limited here because the children had autism spectrum disorders. In an earlier study using SNP arrays [93], rare CNV burden was found to predict intelligence in a small clinical sample. This association was not replicated in two larger, nonclinical samples [94,95]. Intellectual disability, which is excluded from most studies of IQ in the normal range (but see [96]), has been linked to new CNVs on several occasions [35,60,97]. Rauch et al. [35] estimated new SNPs to explain up to 55% of cases of non-syndromic, sporadic intellectual disability in a small exome-sequencing study.

4. The Present Study

We addressed several of the limitations of prior studies in a large, population-based twin and family sample. To isolate the effect of new mutations from the expected, inherited trait level, we controlled for parental intelligence or parental personality traits when assessing the influence of paternal age on these traits in the offspring. We also controlled for birth order, which was correlated with paternal age, to account for the possibility of diminishing parental investment in later-born children [98,99]. We did not need to exploit the genetic similarity of twins for the purposes of our analysis. Instead we used one randomly chosen co-twin from each pair to replicate our results. Thus we always report two coefficients pertaining to twins. Samples with detailed parental and offspring trait measurements such as this one are valuable but rare, which offsets potential problems with generalizability to singletons [100,101].

On theoretical grounds and based on previous results, we predicted a small remaining negative paternal age association with offspring intelligence after applying these controls. We also looked for paternal age associations with offspring head circumference as a proxy for brain size [20,102–104]. Head circumference is highly heritable [105], but not highly correlated (about .10–.20 [19,103]) with intelligence. Because the anatomical and developmental constraints acting on head and brain size imply a buffering against mutation to be adaptive, we did not expect to find a paternal age association with head circumference.

For personality traits, on the other hand, we did not expect to replicate the association between paternal age and offspring externalising behaviour and social functioning reported by Saha et al. [87] and Weiser et al., [86] when using analogue personality traits and controlling for parental personality trait levels. In these studies proxy variables for the parental trait levels were not controlled. Therefore it cannot be ruled out that parental personality affected reproductive timing [90], which could have introduced a spurious association between paternal age and the children's personality. Absence of association after control would be consistent with the theoretical prediction that personality traits are mostly not under mutation-selection balance [9], though it would not provide direct evidence for the absence of mutation-selection balance.

Methods

1. Sample

The sample comprised 1,898 pairs of same-sex twins (52% female; 64% monozygotic) and their parents who participated in the intake assessment of the Minnesota Twin Family Study

(MTFS), an ongoing population-based longitudinal study. State birth records provided the starting point to locating more than 90% of all Minnesotan same-sex twins born in the target periods spanning 1971 to 1994. Twins with birth defects and major disabilities were screened out of the sample. Less than 20% of the located families declined participation. Based on a brief survey which 80% of the decliners completed, it was possible to show that decliners were only slightly less educated (<0.3 years) and did not differ from participants with regard to self-reported mental health. At intake two thirds of the assessed twins were approximately 11 years old (born 1977–1994) and one third were approximately 17 years old (born 1972–1979). Like the population of Minnesota in the periods of their births, the twins predominantly (over 95%) had European ancestry. Iacono, Carlson, Taylor, Elkins, and McGue [106] and Iacono and McGue [107] described the recruitment process and the characteristics of the sample in more detail. The 11-year-old cohort was enriched for twins showing antisocial behaviours by recruiting pairs in which at least one showed symptoms of attention-deficit-hyperactivity disorder or conduct disorder [108]. About 11% of participants were recruited in this way; we refer to them as the "enrichment sample". Neither attention-deficit-hyperactivity-disorder [109] nor conduct disorder [110] has been linked to paternal age.

2. Ethics Statement

The University of Minnesota's institutional review board approved the collection of the data used in this study. Twins gave written informed assent and parents gave written informed consent.

3. Measures

Twins' birth dates were available from state records. Their zygosity was assessed based on the consensus of several methods and serological analyses in case of disagreement. In the intake phone survey, the mother reported the father's birth date and education, the twins' birth weight, any birth complications and whether the twin birth had been full-term or by how many weeks it had been early or late. If the father had taken part in the intake assessment, we used his self-reported birthdate and education data instead. We considered using the mother's report on how many weeks the birth had been early or late to derive the paternal age at conception, but decided against it because the information on gestational age was often missing and the computed paternal age at conception correlated perfectly with paternal age at birth.

The 11-year-old-cohort of twins was assessed at intake using an abbreviated Wechsler Intelligence Scale for Children – Revised (WISC–R). It comprised two verbal (Vocabulary and Information) and two performance (Block Design and Picture Arrangement) subtests, which had been selected to maximize the correlation (.94 [111]) with the full WISC–R. The 17-year-old-cohort and the parents were assessed using the same subtests of the Wechsler Adult Intelligence Scale–Revised (WAIS–R). Altogether, 1,531 families had complete IQ data, due mostly to missing paternal data (see Table 1 for *n*s for each family member as applicable for our analyses).

Both age cohorts completed the eleven primary scales of the Multidimensional Personality Questionnaire (MPQ) only after turning seventeen. Their parents completed the questionnaire at intake (*n*s = 1,109 families with complete data for the superfactors, *n* = 1170 for Absorption). The MPQ primary scales can be aggregated into three superfactors (Positive Affectivity, Negative Affectivity, and Constraint) plus an Absorption factor, which measures a person's proneness to experience imaginative and altered states. Positive Affectivity comprises the scales Well-being,

Table 1. Descriptive statistics for main variables.

Variable	Individual	*n*	Mean	*SD*	Range
Paternal age	Twins	1848	30.15	5.54	15–53
Maternal age	Twins	1876	27.90	4.85	16–44
Birth weight (grams)	Twins	3700	2587	563	566–4961
Nr. of older siblings	Twins	3704	0.94	0.93	0–8
Nr. of younger siblings	Twins	3704	0.85	0.92	0–9
Age at IQ testing	Twins	3796	13.68	2.72	11–19
Age at MPQ testing	Twins	2997	18.29	1.70	16–29
Birth year	Father	1860	1952.14	7.46	1925–1977
	Mother	1888	1954.42	6.90	1934–1976
	Twins	3759	1982.26	6.07	1972–1994
IQ	Father	1562	106.53	14.67	61–151
	Mother	1851	102.29	13.44	70–147
	Twins	3749	102.23	13.93	50–156
Education (years)	Father	1859	14.17	2.51	6–26
	Mother	1879	13.97	2.06	6–24
	Twins	3277	7.49	2.75	2–16
Head circumference (cms.)	Father	1362	578.81	17.15	531–640
	Mother	1614	554.36	18.45	455–767
	Twins	2927	548.45	20.81	228–613
MPQ Positive affectivity	Father	1514	120.64	13.10	76–162
	Mother	1735	120.10	13.12	65–164
	Twins	2931	123.17	13.42	64–166
MPQ Negative affectivity	Father	1514	82.17	13.54	41–130
	Mother	1735	80.81	12.89	38–122
	Twins	2931	88.91	14.27	42–147
MPQ Constraint	Father	1514	144.03	14.55	87–186
	Mother	1735	151.18	13.61	100–195
	Twins	2931	134.09	16.14	58–187
MPQ Absorption	Father	1538	38.52	8.60	18–67
	Mother	1762	41.28	9.16	18–69
	Twins	2976	42.70	9.42	18–72

Note. Total N = 1898 families.
MPQ = Multidimensional Personality Questionnaire.
SD = standard deviation.
doi:10.1371/journal.pone.0090097.t001

Social Potency, Achievement and Social Closeness. Negative Affectivity contains the scales Aggression, Alienation, and Stress Reaction. Constraint consists of Control, Traditionalism, and Harm Avoidance [112]. A joint factor analysis by Church [113] of Tellegen's personality model with the popular Big Five model revealed no gaps in coverage of either instrument in comparison with the other.

Head circumference (*n* = 1,225 families) was measured during the intake assessment.

Fathers who did not take the intelligence test (*n* = 336) had been educated fewer years (Cohen's $d = -0.26$, $p < .001$). Their twins had significantly lower IQs (−0.17, $p < .001$), less constraint (−0.06, $p = .045$), less positive (−0.07, $p = .019$) and more negative affectivity (0.10, $p < .001$). Mothers in families with gaps in paternal data also had significantly lower IQs (−0.09, $p = .014$), less constraint (−0.09, $p = .022$) and more negative affectivity (0.09, $p = .016$).

4. Statistical Analyses

We fitted structural equation models (SEMs) using Mplus version 7 [114] with a robust maximum likelihood estimator. Compared to standard multiple regressions, full information maximum likelihood (FIML [115]) allowed us to use all available data as opposed to e.g. just the 64% complete families for the third intelligence model due mostly to missing information on the covariates paternal intelligence and birth complications. (see also Table 1) By using latent variables we were able to estimate comparable regression coefficients, indicating expected change in outcome in standard deviation units per decade of paternal age, across outcomes with different reliabilities.

We fitted two separate but analogous chains of models for intelligence and head circumference in one chain, and MPQ personality traits in the other. In the intelligence models we let the residuals of the verbal subtests Vocabulary and Information and those of the performance subtests Picture Completion and Block Design correlate. We allowed subscale residuals to correlate between the twin-pairs to allow for similarity greater than expected on the basis of the latent factors. We also let the Absorption factor correlate within the superfactors Positive and Negative Affectivity. A simplified model for IQ can be seen in Figure 1.

In the first model, we included methodological controls, namely child's sex and age at testing, to decrease residual variance and increase predictive power, and zygosity to control for correlated prenatal factors. The main predictor was paternal age at birth in days. In the second model we added parental trait levels. From the second intelligence model on we also added parental years of education as auxiliary variables, to improve the FIML estimation of missing intelligence data for parents. To compare our methods with previous studies we estimated models controlling only for either parental education, or intelligence or both. In the third model we added the number of older non-co-twin siblings (i.e. birth order), birth weight and birth complications as further controls.

For all analyses we chose one twin from each pair at random and then replicated the result with the co-twin data, both resulting coefficients are reported for all central results. We also modelled quadratic trends emulating Malaspina et al.'s [76] analyses, and cubic trends for paternal age as suggested by Crow [44].

Furthermore, we examined associations with the primary scales of MPQ personality using multiple regressions and verbal and performance intelligence using a SEM. We ran analyses with and without the enrichment sample as well as split by sex.

Complete, reproducible reports of the analyses have been made available online at http://opensciencefoundation.org/project/wLrZF/wiki/home.

Results

1. Sensitivity Analysis

Including the enrichment sample made some results reach the conventional level of statistical significance but did not change the pattern of results in a noteworthy manner, so we opted for including it to reach higher power. We did a power analysis with G*Power 3 [116] to compute our study's sensitivity at a power of 95% and a Type I error probability of 5% to estimate the upper-bound effect size that could be detected. Our sensitivity analysis using the *n*s of complete cases indicated that we would be able to find paternal age effects if they explained at least 0.85% of the variance of IQ, 1.06% of head circumference variance, and 1.30% of MPQ personality superfactor score variance. Sensitivity in the FIML analyses would be higher, though we did not perform the simulations necessary to give a precise estimate.

2. Descriptive Statistics and Model Fit

The average paternal age at twin birth was 30.15 years (*SD* = 5.54, range = 15–53) and fathers were born between 1925 and 1977. Mothers were born about 2 years later on average and twins were born on average in 1982. Mothers reported birth complications for 51% of all twin births and an average birth weight of 2587 grams (*SD* = 563). Twins averaged slightly less than one older and one younger sibling, though few had both older and younger siblings. Parents averaged about 2 years of post-high school education and IQs very slightly above average; twin IQs were similar. Descriptive statistics for the other main variables can be found in Table 1. Parent-offspring correlations for IQ (*r*s = .39) and head circumference (*r*s = .24–.28) were moderate but somewhat lower for MPQ personality (*r*s = .10–.21). Correlations between mothers' and fathers' traits were similar (IQ: *r* = .34; MPQ: *r*s = .15–.21), except for head circumference, which was effectively zero (*r* = .04).

Model fit according to root-mean-square error of approximation (RMSEA; both for baseline and full model) and standardized

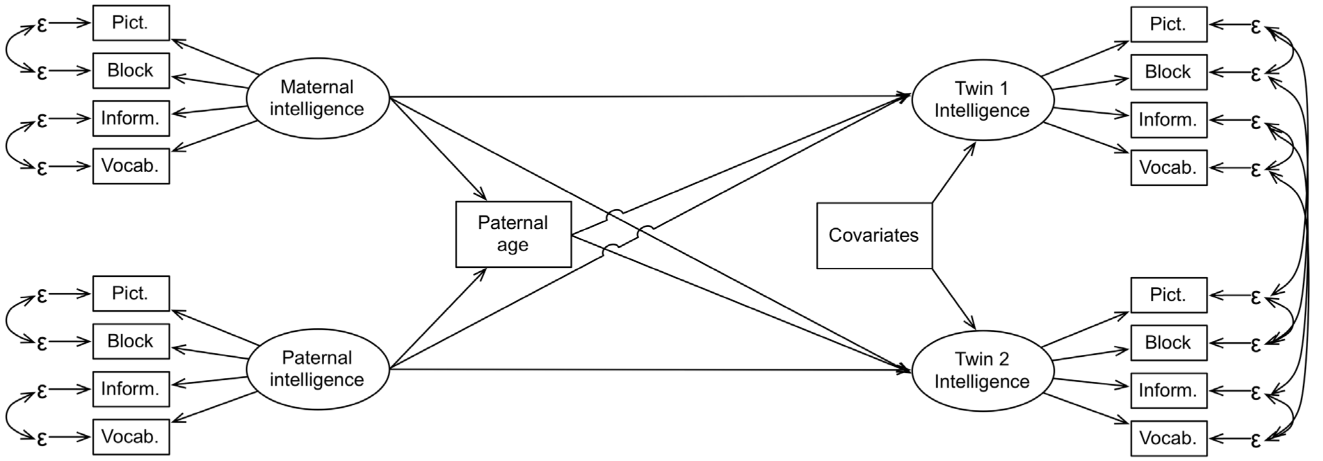

Figure 1. Simplified schematic of the structural equation models for intelligence. Other models were largely analogous, exceptions are explained in the text.
doi:10.1371/journal.pone.0090097.g001

root-mean-square residual (SRMR) are reported in Table 2. Model fit according to χ^2 was always violated owing to the large sample. The measures of close fit for the intelligence models exceeded recommendations by Browne and Cudeck [117]. The MPQ models' fit can still be regarded as reasonable for a parsimonious model because we did not model cross-loadings that were not part of the theoretical factor structure.

3. Main Results

Overall, we found no robust evidence for paternal age associations with offspring intelligence, personality or head circumference (see Table 3 and Figure 2). The regression coefficient of paternal age on offspring intelligence turned from significantly positive to non-significantly negative after controlling for parental intelligence, because both predictor and outcome correlated positively with parental intelligence. The change from b = 0.11 (95% CI [0.06, 0.17]) in the first model to b = −0.04 (95% CI [−0.09, 0.01]) was significant. The confidence intervals overlapped if we controlled for years of education instead of intelligence in the second model (b = 0.04, 95% CI [−0.01, 0.09], see also Figure 3). Controlling for both parental education and intelligence yielded a descriptively larger change than controlling for either one in the regression weight that reached conventional significance (b = −0.06, 95% CI [−0.11, −0.01]). In the third model, correcting for birth order decreased the relation to statistical non-significance (tested by omitting other covariates post-hoc). The regression weights for birth order were −0.15 ($p<.001$, 95% CI [−0.20, −0.10]) and −0.11 for the co-twins ($p<.001$, 95% CI [−0.16, −0.06]).

We also found a positive relation between paternal age and Absorption that was marginally significant for one set of twins (p = 0.079) and conventionally significant (p = 0.038) for the other set of twins in the third model, but not without the enrichment sample. We found no statistically significant relation of paternal age with the MPQ superfactors or with head circumference.

We tested for confounding relations between paternal age and parental traits. In multiple regressions estimated in the SEMs we found positive regression coefficients for maternal ($b = 0.17$, $p<.001$, 95% CI [0.11, 0.22]) and paternal intelligence ($b = 0.15$, $p<.001$, 95% CI [0.09, 0.20]) on paternal age, indicating later reproduction among more intelligent parents. Parental MPQ personality was also related to paternal age. Paternal positive ($b = -0.10$, $p = .013$, 95% CI [−0.19, −0.02]) and negative ($b = -0.12$, $p = .009$, 95% CI [−0.21, −0.03]) affectivity were related to lower paternal age. Other non-significant, but not significantly less important predictors in this regression were maternal negative affectivity ($b = -0.06$, $p = .068$, 95% CI [−0.11, 0.00]) and paternal constraint ($b = 0.06$, $p = .137$, [−0.02, 0.13]). Parental intelligence explained significantly more variance in paternal age ($R^2 = 0.07$, 95% CI [0.05, 0.10]) than parental MPQ personality ($R^2 = 0.02$, 95% CI [0.00, 0.04]). Parental education also predicted paternal age (maternal $b = 0.15$, 95% CI [0.10, 0.21], $p<.001$; paternal $b = 0.06$, 95% CI [−0.00, 0.12], $p = .048$), though the parents' intelligence explained more variance on its own than their education ($R^2 = 0.04$, 95% CI [0.02, 0.05]). Together they did not account for significantly more variance ($R^2 = 0.08$, 95% CI [0.05, 0.10]). In a joint regression on paternal age estimated as part of the second model, the coefficients for maternal intelligence, paternal intelligence, maternal and paternal education respectively were 0.15 ([0.07, 0.23], $p<.001$), 0.16 ([0.07, 0.26], $p = .001$), 0.06 ([−0.01, 0.12], $p = .125$) and −0.07 ([−0.15, 0.01], $p = .096$).

Examining quadratic and cubic effects of paternal age, as well as tests at the subtest and primary scale level, did not yield many noteworthy deviations from the general pattern of non-robust or insignificant associations. Modelling verbal and performance intelligence separately in the second model showed mostly overlapping 95% CIs of [−0.11, 0.00] for verbal and [−0.10, 0.03] for performance intelligence. In the third model we found a quadratic association (linear $b = -0.04$, p = .148, 95% CI [−0.09, 0.01]; quadratic $b = 0.07$, $p = .010$, 95% CI [0.02, 0.12]) of paternal age with offspring verbal IQ that replicated for the co-twins (linear $b = -0.05$, p = .055, 95% CI [−0.11, 0.00]; quadratic $b = 0.07$, $p = .008$, 95% CI [0.02, 0.13]), but it was in the opposite direction of what we had predicted. We tested our results' robustness to leaving out covariates and other modelling decisions such as using FIML instead of multiple imputation, or imposing measurement invariance according to Raykov et al. [118]. With the exception of the aforementioned covariates birth order and parental traits in the intelligence models, this did not lead to changes in the paternal age effect size estimates.

Table 2. Fit indices for the reported models.

Model	*N*	χ^2	*df*	RMSEA [90% CI]	SRMR
Intelligence					
1.	1898	214.46	47	0.04 [0.04, 0.05]	0.03
2.	1898	822.31	199	0.04 [0.04, 0.04]	0.04
3.	1898	972.99	271	0.04 [0.03, 0.04]	0.04
Personality					
1.	1872	3416.21	257	0.08 [0.08, 0.08]	0.08
2.	1886	7388.16	999	0.06 [0.06, 0.06]	0.06
3.	1898	8505.10	1222	0.06 [0.06, 0.06]	0.06

Note. All reported χ^2 were significant ($p<.001$).
df = Degrees of freedom.
RMSEA = Root mean squared error of approximation.
SRMR = Standardized root mean residual.
CI = Confidence interval.
Model 1: Paternal age, twin's age at testing, sex, zygosity.
Model 2: As model 1, plus mother's trait level, father's trait level.
Model 3: As model 2, plus number of older siblings/birth order, birth weight, birth complications.
doi:10.1371/journal.pone.0090097.t002

Discussion

We did not find support for our hypothesis that higher paternal age at offspring conception, as an indicator of more new, harmful mutations, would predict lower offspring intelligence. A small positive association between paternal age and offspring intelligence turned significantly negative after controlling for parental intelligence and education, but this finding was not robust to adding birth order as a covariate, leaving out the enrichment sample, or informally correcting for multiple testing. We found small positive relations between parental intelligence and both paternal and maternal ages, plausibly indicating delayed reproduction among higher-IQ parents. Unlike Rodgers et al. [15] and Neiss et al. [119], who reported that education mediated the relation between maternal intelligence and female age at first birth, we found that parental education did not account for a significant amount of variance in paternal age over and above parental intelligence. This might indicate that paternal and maternal ages at twin birth were not representative of maternal age at first birth (the most commonly used indicator of reproductive timing). We think it is unlikely that this discrepancy reflects deeper underlying differences with regard to reproductive planning in our twin sample as twin births are not usually planned. Differential utilisation of assisted

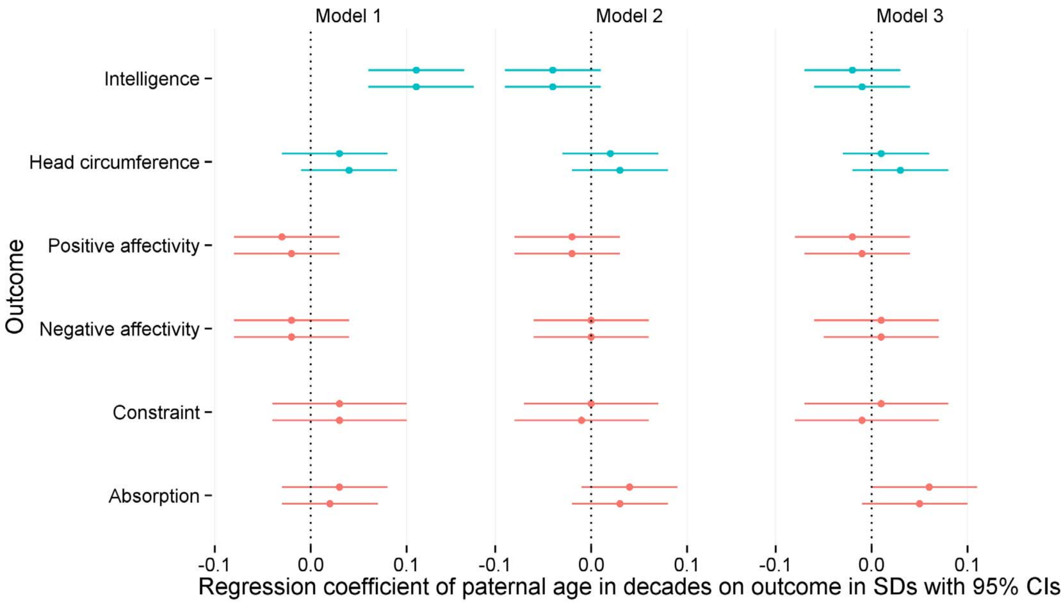

Figure 2. Regression coefficients of paternal age on main outcomes plotted for the two model chains. Same colours indicate coefficients estimated in joint models. Twins are presented separately, with the first co-twins presented first. CI = confidence interval, SD = standard deviation.
doi:10.1371/journal.pone.0090097.g002

reproductive technologies (ART) would further complicate the picture, MTFS twins, however, were born at a time when ART were less common reasons for multiple births [120,121]. Unlike Malaspina et al. [76] we did not find a stronger effect on non-verbal than verbal intelligence. Confidence intervals for the two outcomes strongly overlapped, as they appear to have done in Malaspina et al.'s results as well.

Unexpectedly, we found an association between paternal age and one MPQ scale, Absorption, which was marginally significant for one twin and conventionally significant for the co-twins. To the extent this association might be real, we speculate that it might reflect the well-replicated association of paternal age with offspring schizophrenia, because Absorption has been found to correlate with clinically aberrant experience [122], hallucinations [123] and Psychoticism [124]. Although a potential link with new mutations, as indicated by the parental age association, could explain why Absorption has not been found to be elevated in mostly non-offspring kin of schizophrenia patients [125], we would only

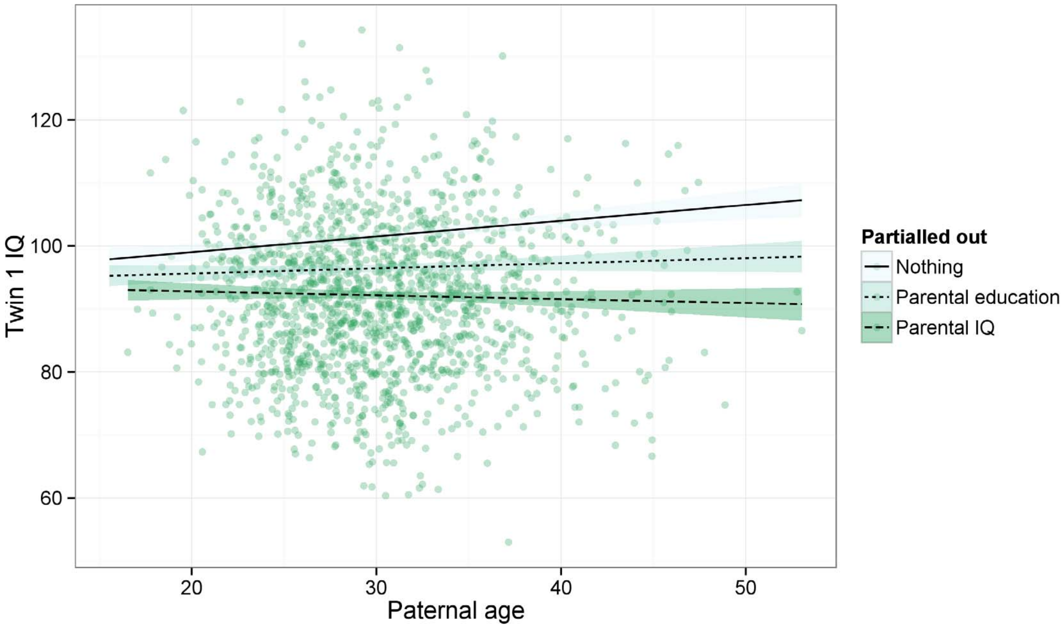

Figure 3. Raw data of the association between paternal age and offspring IQ (only complete cases). Superimposed are three fit lines, with different covariates partialled out of paternal age.
doi:10.1371/journal.pone.0090097.g003

Table 3. Standardized regression coefficients for paternal age in three models.

	Model 1		Model 2		Model 3	
**Variable (each twin)**	***b***	**[95% CI]**	***b***	**[95% CI]**	***b***	**[95% CI]**
Full IQ[a]	0.11***	[0.06, 0.17]	−0.04†	[−0.09, 0.01]	−0.01	[−0.06, 0.04]
[b]	0.11***	[0.06, 0.16]	−0.04	[−0.09, 0.01]	−0.02	[−0.07, 0.03]
Head circumference[a]	0.04	[−0.01, 0.09]	0.03	[−0.02, 0.08]	0.03	[−0.02, 0.08]
[b]	0.03	[−0.03, 0.08]	0.02	[−0.03, 0.07]	0.01	[−0.03, 0.06]
Positive affectivity[a]	−0.02	[−0.08, 0.03]	−0.02	[−0.08, 0.03]	−0.01	[−0.07, 0.04]
[b]	−0.03	[−0.08, 0.03]	−0.02	[−0.08, 0.03]	−0.02	[−0.08, 0.04]
Negative affectivity[a]	−0.02	[−0.08, 0.04]	0.00	[−0.06, 0.06]	0.01	[−0.05, 0.07]
[b]	−0.02	[−0.08, 0.04]	0.00	[−0.06, 0.06]	0.01	[−0.06, 0.07]
Constraint[a]	0.03	[−0.04, 0.10]	−0.01	[−0.08, 0.06]	−0.01	[−0.08, 0.07]
[b]	0.03	[−0.04, 0.10]	0.00	[−0.07, 0.07]	0.01	[−0.07, 0.08]
Absorption[a]	0.02	[−0.03, 0.07]	0.03	[−0.02, 0.08]	0.05†	[−0.01, 0.10]
[b]	0.03	[−0.03, 0.08]	0.04	[−0.01, 0.09]	0.06*	[0.00, 0.11]

Note. Latent variables were standardized. Coefficients (*b*) are the change in outcome in standard deviation units per decade of paternal age. No adjustment of significance levels for multiple testing.
Model 1: Paternal age, twin's age at testing, sex, zygosity.
Model 2: As model 1, plus mother's trait level, father's trait level.
Model 3: As model 2, plus number of older siblings/birth order, birth weight, birth complications.
[a]Twin 1;
[b]Twin 2.
CI = Confidence interval.
†$p<.10$.
*$p<.05$.
**$p<.01$.
***$p<.001$.
doi:10.1371/journal.pone.0090097.t003

cautiously interpret this finding in the light of the fact that the association was not robustly significant.

For the MPQ superfactors, constraint, positive and negative affectivity, we did not find any significant relations between offspring traits and paternal age, either before or after controlling for parental personality. Offspring head circumference was not significantly related to paternal age either. These results provide indirect support for our hypothesis that genetic variance in neither personality traits nor head circumference is under mutation-selection balance [8,9,20].

One of the primary strengths of this study was our ability to control for parental trait levels measured with the same precision as offspring traits when using paternal age as an indicator for likelihood of new mutations. Even though our sample was smaller than those of preceding studies, we would have been able to detect some of the previously reported effect sizes had they been present (e.g. Malaspina et al.'s 2% incremental variance explained [76]), so these early reports may have overestimated the effect size. However, some reported effects would probably (variance explained was rarely reported in previous studies) have been too small for us to detect and we cannot have too much confidence in power estimates derived from previous studies that probably suffered from varying degrees of omitted variable bias. Most importantly, neither the relation with intelligence nor the relations with constraint, positive or negative affectivity were significant. Lack of constraint coupled with negative affectivity is similar to externalising behaviour [126], which Saha et al. [87] reported to be related to paternal age. Positive and negative affectivity are related to social functioning [127], which Weiser et al. [86] found to be associated with paternal age. Despite the smaller size of our sample, we were able estimate the upper effect size boundary when controls for parental trait levels were in place, and can say with some confidence that true effects would not explain more than 1.3% of variance.

With samples in which less variation is accounted for by non-genetic components (e.g. shared-environment), we would expect a paternal age effect attributable to mutations to explain more variation and thus to be more easily detected. This could for example be the case in samples with older offspring [128,129]. However, higher heritability does not imply that it will be easy to detect individual causal genes.

A large effect of paternal age on intelligence would have been consistent with a detrimental burden of new mutations coming from older fathers and would have thus raised the question why selection has not led to early reproduction (or even "andropause", i.e. a complete cessation of male reproductive ability in late life) in men. It would also have indicated selective pressure for transcription accuracy. Very small effects of paternal age are consistent [51] with the hypothesis that new mutations affecting fitness are rare and have small effects on the population level (though their effects on single individuals might still be substantial).

A link between paternal age and a trait in which variation is maintained through mutation-selection balance should persist or even emerge only after controlling for parental trait levels. Parents' intelligence and personality may influence both their reproductive timing and their children's traits, thus constituting an unobserved common cause of both paternal age and offspring traits. If we assume that the mean time at which the parents had the twins was representative of their mean overall reproductive timing (we were unable to test this beyond showing that parental intelligence was unrelated to twins' number of older or younger siblings), parents with higher IQs delayed reproduction compared with those with

lower IQs in our sample. This is the most likely reason that the association between paternal age and offspring intelligence turned from positive to negative when controlling for parental intelligence, an effect that was not apparent when controlling only for parental education, as has been done in previous studies. Because controlling for education led to regression coefficients whose confidence intervals overlapped with those of the first and second models' and because our results regarding the importance of education to reproductive timing differed from previous studies' [15,119], we recommend controlling for both in samples in which fertility may be influenced by personality [4,130,131] and intelligence [15]. In particular, associations with reproductive timing have not yet been demonstrated in a sufficiently wide range of samples which differ with regard to family planning. At least in our sample we did not find any noteworthy changes in the regression coefficients of paternal age on personality when adding parental trait levels as covariates. Thus, it may be possible to assess effects of paternal age on personality in simple cross-sectional samples without having to account for the indirect path through the common cause parental personality.

An interaction between societal factors leading to delayed reproduction and IQ might explain that results differed in previous studies, though they used similar controls and methods. In the case of Auroux et al. [83,84] a largely overlapping research group working with French military recruit data found a negative effect of increasing paternal age on IQ, but could not replicate it in more recent data. If the societal trend towards delayed reproduction in industrialised countries [132] were accelerated in people with higher IQs, parental IQ, as an unobserved common cause in previous studies, would have suppressed the path from paternal age to offspring IQ more in studies of more recent lower-fertility cohorts. Saha et al. [78] and Malaspina et al. [76], who reported a negative association between paternal age and offspring IQ, analysed samples from populations with high average fertility. Average fertility rates in the USA and Israel were 3.6 and 3.8, roughly double those in France, Sweden, the United Kingdom and Minnesota (1.6 to 2.2; national fertility rates at the time of data collection from [133]; Minnesota fertility rates from [134]) in the respective birth cohorts of the studies that did not report negative associations [80,83,85]. Speculatively, any bias resulting from an effect of paternal IQ on both reproductive timing and offspring traits may have differed between these higher- and lower-fertility populations. Thus, societal fertility trends might account for differences among the studies in these different populations.

Limitations

Our sample size was smaller than those of most previous studies (a fourth of Auroux et al.'s [83], a hundredth of Svensson et al.'s [80]), therefore our power to detect effects that explain less than 0.85% of variance was severely restricted. That our IQ tests were more established and comprehensive than the military aptitude tests and school grades used before can only partly compensate this. Because we analysed a rather small and homogeneous sample, our considerations regarding societal trends have to remain speculative and generalizability of results might be restricted. We also cannot know for sure whether paternal age at twin birth was representative of average reproductive timing and whether the associations we report would be replicated for single births. There is evidence against consequential mean differences in the outcomes of interest [100,135]. The relation between advanced maternal age and dizygotic twinning [136] would not, on its own, jeopardise our conclusions, though replication in a singleton sample would of course strengthen our confidence in them. The systematic differences we found for families whose fathers did not participate in the intake assessments may have decreased our chance to find significant results.

Our relative ability to detect any paternal age effects on MPQ personality as opposed to effects on intelligence may have been even lower than indicated by our sensitivity analyses, because we had less MPQ personality data, poorer model fit and less auxiliary information to estimate our models with missing data.

Because major disabilities and birth defects were thoroughly screened out of our sample, our conclusions are limited to intelligence variation in the normal range. Previous studies also conducted their analyses on either nonclinical or clinical samples, but not both. If paternal age were related to intellectual disability, but not intelligence in the normal range, effect sizes would also vary across studies according to the thoroughness of the screening procedure. The mean and variance of paternal age in our sample were similar to previous studies, but we cannot rule out that a larger number of older fathers would have boosted our explanatory power, especially if the effect were exponential.

We may also have omitted important confounding variables. Unlike previous researchers we decided against controlling for maternal age because this would have introduced high multicollinearity with paternal age ($r = 0.80$) and birth order ($r = 0.29$) and led to convergence failures. Findings of an offspring IQ increase with advancing maternal age largely relied on child-rearing, maternal social background and parental psychological adjustment as mediators [137,138], for which we tried to account using parental IQs instead. Socioeconomic status was not controlled either, because we believed controls for intelligence and education to be sufficient. Positive effects of advancing maternal age, if not sufficiently controlled in our study, would have decreased our ability to identify a purported effect of advanced paternal age.

Considerations for Future Studies

We hope future research on paternal age effects on intelligence will benefit from the debate about the effect of birth order on intelligence [98,99,139]. It seems possible to disentangle birth order and paternal age, because they generally have only moderate correlation across families. Some interpretations of the birth order variable (e.g. tutoring by siblings, or decreased paternal investment when multiple children are born in short intervals) would not be consistent with an effect of accumulated germline mutations, but e.g. decreased paternal investment in later-born siblings would be. Many of the challenges that emerged in birth order research apply to paternal age research as well. One example is the debate over whether birth order is also related to decreased intelligence within families [98]. If constant differences between families (e.g. parental intelligence) which are related to both their reproductive decision-making and mean offspring intelligence drive paternal age effects, they would be found between families, but not within. Such effects would not be indicative of new mutations and thus spurious in the context of our research question.

Of course within-family findings are not beyond reproach either [140]. For example, families may decide to have more children after their economic situations improve, allowing them to provide better environments for their later-borns. Additionally, within-family research may suffer from limited variance in paternal age, because most women do not have children across their whole reproductive lifespan in industrialised countries [141] and because fathers can only have children across their whole reproductive

lifespans if they find younger partners after their original partner has gone into menopause.

Paternal age and birth order have the same rank-order within many "traditional" families, making it very difficult to compare their predictive accuracy within families in all but the largest samples. To break up this confound, the variable birth order could be substituted by direct assessments of the constructs for which it is supposed to be a proxy: differential parental investment, sibling tutoring and so forth.

Similarly, whole-genome- and exome-sequencing studies of families, which allow for counting new mutations by comparing the genomes or exomes of parents and children, have to control the various factors, especially parental trait levels, that might influence reproductive timing and thereby new mutation incidence. For example, Iossifov et al. [91] found that "likely gene-disrupting mutations" predicted autism, but were not related to intelligence in an exome-sequencing study of 343 families with children on the autism spectrum and their unaffected siblings. Inherited subsyndromal autism was an unlikely confound for the autism finding, because they employed a simplex sample (i.e. no relatives with autism spectrum disorders). However, parental intelligence was not controlled. The same concern applies to Sanders et al.'s [58,92] results, which implicated new copy-number but not single nucleotide variants in intelligence in a clinical sample from the same population, the Simons Simplex Collection. Most importantly, the exome constitutes only the coding 1% of the genome; plausibly more polymorphisms affecting complex, continuous traits may be found in the regulatory sequences of the genome [142]. The total contributions of new mutations to intelligence are only beginning to come into the reaches of current molecular genetic methods (especially the still expensive sequencing techniques, see [36,57,73,74]).

Another way paternal age studies can improve their estimates is by considering the insights from Flynn effect research (Flynn [143,144], reviewed by Mingroni [145]). The rise in intelligence test scores over time could mean that older parents in previous studies were also from earlier cohorts with lower test scores. Possibly, their offspring would have lower test scores as well. Malaspina et al. [76] dismissed the Flynn effect as a confound, because it had not been found to occur within families, nor to affect heritability estimates for intelligence. However, the Flynn effect has since been shown in brothers [146]. Johnson, Penke, and Spinath [147] reasoned that high heritability of a trait should not be construed as an argument against environmentally mediated secular increases: Gene-environment interactions may be revealed or hidden, depending on whether the necessary variability in the environment is present. Wicherts et al. [148] showed that measurement invariance of general intelligence was violated with respect to different cohorts, making it unlikely that the observed gains reflected latent "real" increases. Previous studies which used sum scores could not guard against bias resulting from changes in subtest scores rather than general intelligence by checking their results' robustness to imposing measurement invariance.

A paternal age effect could also mask a rise of test scores within families. Rodgers [149] had dismissed both the within-family Flynn and the birth order effect, arguing that neither was present in his data, even though the two effects might have cancelled each other out [145].

In fact, Sundet, Borren, and Tambs [146] have proposed changes in fertility patterns as one cause of the Flynn effect after finding that decreases in the prevalence of large families explain part of the increase in intelligence scores. They examined data on Norwegian conscripts, but they aggregated mean sibling IQ. Plausibly the actual explanatory variable is found elsewhere, at the individual level. A trend towards delayed reproduction in intelligent parents [15] and the general population [132], and thus an increase in new mutations, could be partly culpable for the reports of a slowing [150], stop [151] or even reversal [152,153] of the Flynn effect in Scandinavian countries. We might be able to explain the null effects of paternal age on intelligence in more recent analyses, in which parental intelligence was not controlled [80,83,85] by delayed reproduction among more intelligent parents, though our study raises the question whether any paternal age effect attributable to mutations exists and is substantial. Taking into account these known problems with measuring differences in intelligence over time could serve to improve future research into paternal age.

Additionally, research in more diverse populations is warranted because results from the 1000 Genomes Project Consortium [142] suggest that populations are substantially differentiated geographically with regard to low-frequency variants. The results also suggest differences in strength and efficacy of purifying selection across populations, which are highly relevant to paternal age research.

Future research on paternal age effects may benefit from the history of birth order research and employ controls for parental traits, within-family designs (eliminating between-family confounds) or pedigree analyses of paternal age effects across several generations (ruling out alternative environmental and epigenetic explanations and boosting explained variance [154]) depending on the availability of data.

Conclusions

Controlling for parental trait level, we were unable to show significant effects of paternal age, a proxy for new genetic mutations, on offspring IQ, head circumference, or personality traits. Parents' IQ and personality were correlated with their reproductive timing. This necessitates thorough control of parental trait levels in future studies on paternal age effects. Our sample size was insufficient to reveal very small effects, but our results can be understood as providing an upper boundary of any expected effect sizes. Reported effect sizes of paternal age on offspring personality and intelligence have been heterogeneous. So far no clear picture of the role of mutation-selection balance has emerged from these studies. More research in different populations and converging evidence may enable us to find out more about the evolutionary mechanisms that maintain genetic variance in traits like intelligence. If any paternal age effects on intelligence exist, they are probably very small. Narrowing down the precise effect size and ruling out the many possible confounds would be steps towards quantifying the contribution of de novo mutation-selection balance to intelligence and other individual differences. If other studies show paternal age effects on intelligence to be negligible but confirm the link between paternal age and de novo mutations, this prompts interesting research questions into the robustness of the highly polygenic intelligence trait.

Acknowledgments

We would like to thank Roos Hutteman and Sarah J. Lennartz for providing helpful feedback on an earlier version of this manuscript.

Author Contributions

Conceived and designed the experiments: RCA LP. Performed the experiments: WGI MM. Analyzed the data: RCA. Contributed reagents/materials/analysis tools: WJ WGI MM. Wrote the paper: RCA LP WJ WGI MM.

References

1. Roberts BWB, Kuncel N, Shiner R, Caspi A, Goldberg LR (2007) The power of personality: The comparative validity of personality traits, socio-economic status, and cognitive ability for predicting important life outcomes. Perspect Psychol Sci 2: 313–345. doi:10.1111/j.1745-6916.2007.00047.x.
2. Deary IJ, Whiteman MC, Starr JM, Whalley LJ, Fox HC (2004) The impact of childhood intelligence on later life: following up the Scottish Mental Surveys of 1932 and 1947. J Pers Soc Psychol 86: 130–147. doi:10.1037/0022-3514.86.1.130.
3. Whalley LJ, Deary IJ (2001) Longitudinal cohort study of childhood IQ and survival up to age 76. Br Med J 322: 819–819. doi:10.1136/bmj.322.7290.819.
4. Hutteman R, Bleidorn W, Penke L, Denissen JJA (2012) It takes two: A longitudinal dyadic study on predictors of fertility outcomes. J Pers: 1–12. doi:10.1111/jopy.12006.
5. Jokela M, Hintsa T, Hintsanen M, Keltikangas-Järvinen L (2010) Adult temperament and childbearing over the life course. Eur J Pers 166: 151–166. doi:10.1002/per.749.
6. Jokela M, Kivimäki M, Elovainio M, Keltikangas-Järvinen L (2009) Personality and having children: a two-way relationship. J Pers Soc Psychol 96: 218–230. doi:10.1037/a0014058.
7. Von Stumm S, Batty GD, Deary IJ (2011) Marital status and reproduction: Associations with childhood intelligence and adult social class in the Aberdeen children of the 1950s study. Intelligence 39: 161–167. doi:10.1016/j.intell.2011.02.007.
8. Penke L (2010) Bridging the gap between modern evolutionary psychology and the study of individual differences. In: Buss DM, Hawley PH, editors. The evolution of personality and individual differences. New York: Oxford University Press. 243–279.
9. Penke L, Denissen JJA, Miller GF (2007) The evolutionary genetics of personality. Eur J Pers 21: 549–587. doi:10.1002/per.629.
10. Buss DM (1989) Sex differences in human mate preferences: Evolutionary hypotheses tested in 37 cultures. Behav Brain Sci 12: 1–49. doi:10.1017/S0140525X00023992.
11. Li NP, Bailey JM, Kenrick DT, Linsenmeier JAW (2002) The necessities and luxuries of mate preferences: Testing the tradeoffs. J Pers Soc Psychol 82: 947–955. doi:10.1037/0022-3514.82.6.947.
12. Miller GF (2000) Sexual selection for indicators of intelligence. In: Bock J, Goode J, Webb K, editors. The Nature of Intelligence. New York: Wiley. 260–275.
13. Stone EA, Shackelford TK, Buss DM (2012) Is variability in mate choice similar for intelligence and personality traits? Testing a hypothesis about the evolutionary genetics of personality. Intelligence 40: 33–37. doi:10.1016/j.intell.2011.10.003.
14. Udry JR (1978) Differential fertility by intelligence: The role of birth planning. Soc Biol 25: 10–14. doi:10.1080/19485565.1978.9988313.
15. Rodgers JL, Kohler H-P, McGue M, Behrman JR, Petersen I, et al. (2008) Education and cognitive ability as direct, mediating, or spurious influences on female age at first birth: Behavior genetic models fit to Danish twin data. Am J Sociol 114: 202–232. doi:10.1086/592205.
16. Agrawal AF, Whitlock MC (2011) Mutation load: The fitness of individuals in populations where deleterious alleles are abundant. Annu Rev Ecol Evol Syst 43: 115–135. doi:10.1146/annurev-ecolsys-110411-160257.
17. Bates TC (2007) Fluctuating asymmetry and intelligence. Intelligence 35: 41–46. doi:10.1016/j.intell.2006.03.013.
18. De Visser JAGM, Hermisson J, Wagner GP, Ancel Meyers L, Bagheri-Chaichian H, et al. (2003) Perspective: Evolution and detection of genetic robustness. Evolution 57: 1959–1972. doi:10.1554/02-750R.
19. Rushton JP, Ankney CD (2009) Whole brain size and general mental ability: a review. Int J Neurosci 119: 691–731. doi:10.1080/00207450802325843.
20. Miller GF, Penke L (2007) The evolution of human intelligence and the coefficient of additive genetic variance in human brain size. Intelligence 35: 97–114. doi:10.1016/j.intell.2006.08.008.
21. Hughes KA, Burleson MH (2000) Evolutionary causes of genetic variation in fertility and other fitness components. In: Rodgers JL, Rowe DC, Miller WB, editors. Genetic Influences on Human Fertility and Sexuality. Boston, MA: Springer US. 7–33. Available: http://www.springerlink.com/index/10.1007/978-1-4615-4467-8_3. Accessed 15 July 2013.
22. Benyamin B, St. Pourcain B, Davis OS, Davies G, Hansell NK, et al. (2013) Childhood intelligence is heritable, highly polygenic and associated with FNBP1L. Mol Psychiatry. doi:10.1038/mp.2012.184.
23. Chabris CF, Hebert BM, Benjamin DJ, Beauchamp J, Cesarini D, et al. (2012) Most reported genetic associations with general intelligence are probably false positives. Psychol Sci 23: 1314–1323. doi:10.1177/0956797611435528.
24. Davies G, Tenesa A, Payton A, Yang J, Harris SE, et al. (2011) Genome-wide association studies establish that human intelligence is highly heritable and polygenic. Mol Psychiatry 16: 996–1005. doi:10.1038/mp.2011.85.
25. Plomin R, Haworth CMA, Meaburn EL, Price TS, Wellcome Trust Case Control Consortium 2, et al. (2013) Common DNA markers can account for more than half of the genetic influence on cognitive abilities. Psychol Sci 24: 562–568. doi:10.1177/0956797612457952.
26. Verweij KJHH, Yang J, Lahti J, Veijola J, Hintsanen M, et al. (2012) Maintenance of genetic variation in human personality: testing evolutionary models by estimating heritability due to common causal variants and investigating the effect of distant inbreeding. Evolution 66: 3238–3251. doi:10.1111/j.1558-5646.2012.01679.x.
27. Vinkhuyzen AAE, Pedersen NL, Yang J, Lee SH, Magnusson PKE, et al. (2012) Common SNPs explain some of the variation in the personality dimensions of neuroticism and extraversion. Transl Psychiatry 2: e102. doi:10.1038/tp.2012.27.
28. Anderson CA, Soranzo N, Zeggini E, Barrett JC (2011) Synthetic associations are unlikely to account for many common disease genome-wide association signals. PLoS Biol 9: e1000580. doi:10.1371/journal.pbio.1000580.
29. Dickson SP, Wang K, Krantz I, Hakonarson H, Goldstein DB (2010) Rare variants create synthetic genome-wide associations. PLoS Biol 8: e1000294. doi:10.1371/journal.pbio.1000294.
30. Mitchell KJ (2012) What is complex about complex disorders? Genome Biol 13: 237. doi:10.1186/gb-2012-13-1-237.
31. McClellan J, King M-C (2010) Genetic Heterogeneity in Human Disease. Cell 141: 210–217. doi:10.1016/j.cell.2010.03.032.
32. Agarwala V, Flannick J, Sunyaev S, GoT2D Consortium, Altshuler D (2013) Evaluating empirical bounds on complex disease genetic architecture. Nat Genet advance online publication. Available: http://www.nature.com/ng/journal/vaop/ncurrent/full/ng.2804.html. Accessed 28 October 2013.
33. Gibson G (2011) Rare and common variants: twenty arguments. Nat Rev Genet 13: 135–145. doi:10.1038/nrg3118.
34. Plomin R, Spinath FM (2004) Intelligence: genetics, genes, and genomics. J Pers Soc Psychol 86: 112–129. doi:10.1037/0022-3514.86.1.112.
35. Rauch A, Wieczorek D, Graf E, Wieland T, Endele S, et al. (2012) Range of genetic mutations associated with severe non-syndromic sporadic intellectual disability: an exome sequencing study. Lancet 380: 1674–1682. doi:10.1016/S0140-6736(12)61480-9.
36. Olson MV (2012) Human genetic individuality. Annu Rev Genomics Hum Genet 13: 1–27. doi:10.1146/annurev-genom-090711-163825.
37. De Moor MHM, Costa PT, Terracciano A, Krueger RF, de Geus EJC, et al. (2012) Meta-analysis of genome-wide association studies for personality. Mol Psychiatry 17: 337–349. doi:10.1038/mp.2010.128.
38. Munafò MR, Flint J (2011) Dissecting the genetic architecture of human personality. Trends Cogn Sci 15: 395–400. doi:10.1016/j.tics.2011.07.007.
39. Buss DM (2009) How can evolutionary psychology successfully explain personality and individual differences? Perspect Psychol Sci 4: 359–366. doi:10.1111/j.1745-6924.2009.01138.x.
40. Lukaszewski AW, Roney JR (2011) The origins of extraversion: joint effects of facultative calibration and genetic polymorphism. Pers Soc Psychol Bull 37: 409–421. doi:10.1177/0146167210397209.
41. Miller GF (2007) Sexual selection for moral virtues. Q Rev Biol 82: 97–125. doi:10.1086/517857.
42. Keightley PD (2012) Rates and fitness consequences of new mutations in humans. Genetics 190: 295–304. doi:10.1534/genetics.111.134668.
43. Kong A, Frigge ML, Masson G, Besenbacher S, Sulem P, et al. (2012) Rate of de novo mutations and the importance of father's age to disease risk. Nature 488: 471–475. doi:10.1038/nature11396.
44. Crow JF (1997) The high spontaneous mutation rate: Is it a health risk? Proc Natl Acad Sci 94: 8380–8386. doi:10.1073/pnas.94.16.8380.
45. Crow JF (2000) The origins, patterns and implications of human spontaneous mutation. Nat Rev Genet 1: 40–47. doi:10.1038/35049558.
46. Glaser RL, Broman KW, Schulman RL, Eskenazi B, Wyrobek AJ, et al. (2003) The paternal-age effect in Apert syndrome is due, in part, to the increased frequency of mutations in sperm. Am J Hum Genet 73: 939–947. doi:10.1086/378419.
47. Glaser RL, Jabs EW (2004) Dear old dad. Sci Aging Knowl Environ 2004: 1–11. doi:10.1126/sageke.2004.3.re1.
48. Jones KL, Smith DW, Harvey MAS, Hall BD, Quan L (1975) Older paternal age and fresh gene mutation: Data on additional disorders. J Pediatr 86: 84–88. doi:10.1016/S0022-3476(75)80709-8.
49. Campbell CD, Chong JX, Malig M, Ko A, Dumont BL, et al. (2012) Estimating the human mutation rate using autozygosity in a founder population. Nat Genet 44: 1277–1281. doi:10.1038/ng.2418.
50. Crow JF (2006) Age and sex effects on human mutation rates: an old problem with new complexities. J Radiat Res (Tokyo) 47: 75–82. doi:10.1269/jrr.47.B75.
51. Campbell CD, Eichler EE (2013) Properties and rates of germline mutations in humans. Trends Genet: 1–10. doi:10.1016/j.tig.2013.04.005.
52. Choi S-K, Yoon S-R, Calabrese P, Arnheim N (2008) A germ-line-selective advantage rather than an increased mutation rate can explain some unexpectedly common human disease mutations. Proc Natl Acad Sci U S A 105: 10143–10148. doi:10.1073/pnas.0801267105.
53. Crow JF (2003) There's something curious about paternal-age effects. Science 301: 606–607. doi:10.1126/science.1088552.
54. Tiemann-Boege I, Navidi W, Grewal R, Cohn D, Eskenazi B, et al. (2002) The observed human sperm mutation frequency cannot explain the achondroplasia paternal age effect. Proc Natl Acad Sci U S A 99: 14952–14957. doi:10.1073/pnas.232568699.

55. Yoon S-R, Qin J, Glaser RL, Jabs EW, Wexler NS, et al. (2009) The ups and downs of mutation frequencies during aging can account for the Apert syndrome paternal age effect. PLoS Genet 5: e1000558. doi:10.1371/journal.pgen.1000558.
56. Paul C, Robaire B (2013) Ageing of the male germ line. Nat Rev Urol 10: 227–234. doi:10.1038/nrurol.2013.18.
57. Veltman JA, Brunner HG (2012) De novo mutations in human genetic disease. Nat Rev Genet 13: 565–575. doi:10.1038/nrg3241.
58. Sanders SJ, Murtha MT, Gupta AR, Murdoch JD, Raubeson MJ, et al. (2012) De novo mutations revealed by whole-exome sequencing are strongly associated with autism. Nature 485: 237–241. doi:10.1038/nature10945.
59. Flatscher-Bader T, Foldi CJ, Chong S, Whitelaw E, Moser RJ, et al. (2011) Increased de novo copy number variants in the offspring of older males. Transl Psychiatry 1: e34. doi:10.1038/tp.2011.30.
60. Hehir-Kwa JY, Rodríguez-Santiago B, Vissers LE, de Leeuw N, Pfundt R, et al. (2011) De novo copy number variants associated with intellectual disability have a paternal origin and age bias. J Med Genet 48: 776–778. doi:10.1136/jmedgenet-2011-100147.
61. Curley JP, Mashoodh R, Champagne FA (2011) Epigenetics and the origins of paternal effects. Horm Behav 59: 306–314. doi:10.1016/j.yhbeh.2010.06.018.
62. Croen LA, Najjar DV, Fireman B, Grether JK (2007) Maternal and paternal age and risk of autism spectrum disorders. Arch Pediatr Adolesc Med 161: 334–340. doi:10.1001/archpedi.161.4.334.
63. Perrin MC, Brown AS, Malaspina D (2007) Aberrant epigenetic regulation could explain the relationship of paternal age to schizophrenia. Schizophr Bull 33: 1270–1273. doi:10.1093/schbul/sbm093.
64. Keller MC, Miller G (2006) Resolving the paradox of common, harmful, heritable mental disorders: Which evolutionary genetic models work best? Behav Brain Sci 29. doi:10.1017/S0140525X06009095.
65. Uher R (2009) The role of genetic variation in the causation of mental illness: an evolution-informed framework. Mol Psychiatry 14: 1072–1082. doi:10.1038/mp.2009.85.
66. Miller B, Messias E, Miettunen J, Alaraisanen A, Jarvelin M-R, et al. (2010) Meta-analysis of paternal age and schizophrenia risk in male versus female offspring. Schizophr Bull 37: 1039–1047. doi:10.1093/schbul/sbq011.
67. Böök J (1953) Schizophrenia as a gene mutation. Hum Hered 4: 133–139. doi:10.1159/000150736.
68. Malaspina D, Harlap S, Fennig S, Heiman D, Nahon D, et al. (2001) Advancing paternal age and the risk of schizophrenia. Arch Gen Psychiatry 58: 361.
69. Reichenberg A, Gross R, Weiser M, Bresnahan M, Silverman J, et al. (2006) Advancing paternal age and autism. Arch Gen Psychiatry 63: 1026–1032. doi:10.1001/archpsyc.63.9.1026.
70. Frans EM, Sandin S, Reichenberg A, Lichtenstein P, Långström N, et al. (2008) Advancing paternal age and bipolar disorder. Arch Gen Psychiatry 65: 1034–1040. doi:10.1001/archpsyc.65.9.1034.
71. Lopez-Castroman J, Gómez DD, Belloso JJC, Fernandez-Navarro P, Perez-Rodriguez MM, et al. (2010) Differences in maternal and paternal age between schizophrenia and other psychiatric disorders. Schizophr Res 116: 184–190. doi:10.1016/j.schres.2009.11.006.
72. Laursen TM, Munk-Olsen T, Nordentoft M, Mortensen PB (2007) A comparison of selected risk factors for unipolar depressive disorder, bipolar affective disorder, schizoaffective disorder, and schizophrenia from a Danish population-based cohort. J Clin Psychiatry 68: 1673–1681. doi:10.4088/JCP.v68n1106.
73. Gratten J, Visscher PM, Mowry BJ, Wray NR (2013) Interpreting the role of de novo protein-coding mutations in neuropsychiatric disease. Nat Genet 45: 234–238. doi:10.1038/ng.2555.
74. Muers M (2012) Human genetics: Fruits of exome sequencing for autism. Nat Rev Genet 13: 377. doi:10.1038/nrg3248.
75. Xu B, Ionita-Laza I, Roos JL, Boone B, Woodrick S, et al. (2012) De novo gene mutations highlight patterns of genetic and neural complexity in schizophrenia. Nat Genet: 1–7. doi:10.1038/ng.2446.
76. Malaspina D, Reichenberg A, Weiser M, Fennig S, Davidson M, et al. (2005) Paternal age and intelligence: implications for age-related genomic changes in male germ cells. Psychiatr Genet 15: 117–125. doi:10.1097/00041444-200506000-00008.
77. David AS, Malmberg A, Brandt L, Allebeck P, Lewis G (1997) IQ and risk for schizophrenia: a population-based cohort study. Psychol Med 27: 1311–1323. doi:10.1017/S0033291797005680.
78. Saha S, Barnett AG, Foldi C, Burne TH, Eyles DW, et al. (2009) Advanced paternal age is associated with impaired neurocognitive outcomes during infancy and childhood. PLoS Med 6: e40. doi:10.1371/journal.pmed.1000040.
79. Edwards RD, Roff J (2010) Negative effects of paternal age on children's neurocognitive outcomes can be explained by maternal education and number of siblings. PLoS ONE 5: e12157. doi:10.1371/journal.pone.0012157.
80. Svensson AC, Abel K, Dalman C, Magnusson C (2011) Implications of advancing paternal age: does it affect offspring school performance? PLoS ONE 6: e24771. doi:10.1371/journal.pone.0024771.
81. Whitcomb BW, Schisterman EF, Perkins NJ, Platt RW (2009) Quantification of collider-stratification bias and the birthweight paradox. Paediatr Perinat Epidemiol 23: 394–402. doi:10.1111/j.1365-3016.2009.01053.x.Quantification.
82. Reichman NE, Teitler JO (2006) Paternal age as a risk factor for low birthweight. Am J Public Health 96: 862–866. doi:10.2105/AJPH.2005.066324.
83. Auroux MR, Volteau M, Ducot B, Wack T, Letierce A, et al. (2009) Progeny's mental aptitudes in man: Relationship with parental age at conception and with some environmental factors. C R Biol 332: 603–612. doi:10.1016/j.crvi.2009.02.008.
84. Auroux MR, Mayaux M-J, Guihard-Moscato ML, Fromantin M, Barthe J, et al. (1989) Paternal age and mental functions of progeny in man. Hum Reprod 4: 794–797.
85. Whitley E, Deary IJ, Der G, Batty GD, Benzeval M (2012) Paternal age in relation to offspring intelligence in the west of Scotland Twenty-07 prospective cohort study. PLoS ONE 7: e52112. doi:10.1371/journal.pone.0052112.
86. Weiser M, Reichenberg A, Werbeloff N, Kleinhaus K, Lubin G, et al. (2008) Advanced parental age at birth is associated with poorer social functioning in adolescent males: shedding light on a core symptom of schizophrenia and autism. Schizophr Bull 34: 1042–1046. doi:10.1093/schbul/sbn109.
87. Saha S, Barnett AG, Buka SL, McGrath JJ (2009) Maternal age and paternal age are associated with distinct childhood behavioural outcomes in a general population birth cohort. Schizophr Res 115: 130–135. doi:10.1016/j.schres.2009.09.012.
88. Lundström S, Haworth CMA, Carlström E, Gillberg C, Mill J, et al. (2010) Trajectories leading to autism spectrum disorders are affected by paternal age: findings from two nationally representative twin studies. J Child Psychol Psychiatry 51: 850–856. doi:10.1111/j.1469-7610.2010.02223.x.
89. Robinson EB, Munir K, McCormick MC, Koenen KC, Santangelo SL (2011) Brief report: No association between parental age and extreme social-communicative autistic traits in the general population. J Autism Dev Disord 41: 1733–1737. doi:10.1007/s10803-011-1202-4.
90. Jokela M, Alvergne A, Pollet TV, Lummaa V (2011) Reproductive behavior and personality traits of the Five Factor Model. Eur J Pers 25: 487–500. doi:10.1002/per.822.
91. Iossifov I, Ronemus M, Levy D, Wang Z, Hakker I, et al. (2012) De novo gene disruptions in children on the autistic spectrum. Neuron 74: 285–299. doi:10.1016/j.neuron.2012.04.009.
92. Sanders SJ, Ercan-Sencicek AG, Hus V, Luo R, Murtha MT, et al. (2011) Multiple recurrent de novo CNVs, including duplications of the 7q11.23 Williams syndrome region, are strongly associated with autism. Neuron 70: 863–885. doi:10.1016/j.neuron.2011.05.002.
93. Yeo RA, Gangestad SW, Liu J, Calhoun VD, Hutchison KE (2011) Rare copy number deletions predict individual variation in intelligence. PLoS ONE 6: e16339. doi:10.1371/journal.pone.0016339.
94. Bagshaw ATM, Horwood LJ, Liu Y, Fergusson DM, Sullivan PF, et al. (2013) No effect of genome-wide copy number variation on measures of intelligence in a New Zealand birth cohort. PLoS ONE 8: e55208. doi:10.1371/journal.pone.0055208.
95. McRae AF, Wright MJ, Hansell NK, Montgomery GW, Martin NG (2013) No association between general cognitive ability and rare copy number variation. Behav Genet 43: 202–207. doi:10.1007/s10519-013-9587-9.
96. Johnson W, Carothers A, Deary IJ (2009) A role for the X chromosome in sex differences in variability in general intelligence? Perspect Psychol Sci 4: 598–611. doi:10.1111/j.1745-6924.2009.01168.x.
97. Vissers LELM, de Ligt J, Gilissen C, Janssen I, Steehouwer M, et al. (2010) A de novo paradigm for mental retardation. Nat Genet 42: 1109–1112. doi:10.1038/ng.712.
98. Bjerkedal T, Kristensen P, Skjeret G, Brevik J (2007) Intelligence test scores and birth order among young Norwegian men (conscripts) analyzed within and between families. Intelligence 35: 503–514. doi:10.1016/j.intell.2007.01.004.
99. Kristensen P, Bjerkedal T (2007) Explaining the relation between birth order and intelligence. Science 316: 1717. doi:10.1126/science.1141493.
100. Johnson W, Krueger RF, Bouchard TJ, McGue M (2002) The personalities of twins: just ordinary folks. Twin Res Hum Genet 5: 125–131. doi:10.1375/1369052022992.
101. Bouchard TJ, McGue M (2003) Genetic and environmental influences on human psychological differences. J Neurobiol 54: 4–45. doi:10.1002/neu.10160.
102. Bartholomeusz HH, Courchesne E, Karns CM (2002) Relationship between head circumference and brain volume in healthy normal toddlers, children, and adults. Neuropediatrics 33: 239–241. doi:10.1055/s-2002-36735.
103. Royle NA, Booth T, Valdés Hernández MC, Penke L, Murray C, et al. (2013) Estimated maximal and current brain volume predict cognitive ability in old age. Neurobiol Aging. Available: http://www.sciencedirect.com/science/article/pii/S019745801300225X. Accessed 16 July 2013.
104. Friedman L, Wiechers IR, Cerny CA, Schulz SC, Buckley P (2000) If patients with schizophrenia have small brains, why don't they have small heads? Schizophr Res 42: 1–6. doi:10.1016/S0920-9964(99)00098-5.
105. Smit DJA, Luciano M, Bartels M, van Beijsterveldt CEM, Wright MJ, et al. (2010) Heritability of head size in Dutch and Australian twin families at ages 0–50 years. Twin Res Hum Genet 13: 370–380. doi:10.1375/twin.13.4.370.
106. Iacono WG, Carlson SR, Taylor J, Elkins IJ, McGue M (1999) Behavioral disinhibition and the development of substance-use disorders: Findings from the Minnesota Twin Family Study. Dev Psychopathol 11: 869–900. doi:10.1017/S0954579499002369.

107. Iacono WG, McGue M (2002) Minnesota Twin Family Study. Twin Res Hum Genet 5: 482–487. doi:10.1375/136905202320906327.
108. Keyes MA, Malone SM, Elkins IJ, Legrand LN, McGue M, et al. (2009) The enrichment study of the Minnesota twin family study: increasing the yield of twin families at high risk for externalizing psychopathology. Twin Res Hum Genet 12: 489–501. doi:10.1375/twin.12.5.489.
109. Gabis L, Raz R, Kesner-Baruch Y (2010) Paternal age in autism spectrum disorders and ADHD. Pediatr Neurol 43: 300–302. doi:10.1016/j.pediatrneurol.2010.05.022.
110. Wakschlag LS, Gordon RA, Lahey BB, Loeber R, Green SM, et al. (2000) Maternal age at first birth and boys' risk for conduct disorder. J Res Adolesc 10: 417–441. doi:10.1207/SJRA1004_03.
111. Sattler JM (1974) Assessment of children's intelligence (Revised Reprint). Philadelphia, PA: WB Saunders.
112. Tellegen A, Waller NG (2008) Exploring personality through test construction: Development of the Multidimensional Personality Questionnaire. In: Boyle GJ, Matthews G, Saklofske DH, editors. Handbook of Personality Theory and Testing, Vol. II, Personality Measurement and Assessment. Thousand Oaks, CA: Sage, Vol. 2. 261–292.
113. Church AT (1994) Relating the Tellegen and five-factor models of personality structure. J Pers Soc Psychol 67: 898–909. doi:10.1037/0022-3514.67.5.898.
114. Muthén LK, Muthén BO (1998) Mplus User's Guide. Seventh Edition. Los Angeles, CA: Muthén & Muthén.
115. Enders C, Bandalos D (2001) The relative performance of full information maximum likelihood estimation for missing data in structural equation models. Struct Equ Model Multidiscip J 8: 430–457. doi:10.1207/S15328007SEM0803_5.
116. Faul F, Erdfelder E, Buchner A, Lang A-G (2009) Statistical power analyses using G*Power 3.1: Tests for correlation and regression analyses. Behav Res Methods 41: 1149–1160. doi:10.3758/BRM.41.4.1149.
117. Browne MW, Cudeck R (1992) Alternative ways of assessing model fit. Sociol Methods Res 21: 230–258. doi:10.1177/0049124192021002005.
118. Raykov T, Marcoulides GA, Li C-H (2012) Measurement Invariance for Latent Constructs in Multiple Populations: A Critical View and Refocus. Educ Psychol Meas 72: 954–974. doi:10.1177/0013164412441607.
119. Neiss M, Rowe DC, Rodgers JL (2002) Does education mediate the relationship between IQ and age of first birth? A behavioural genetic analysis. J Biosoc Sci 34: 259–275. doi:10.1017/S0021932002002596.
120. Reynolds MA, Schieve LA, Martin JA, Jeng G, Macaluso M (2003) Trends in multiple births conceived using assisted reproductive technology, United States, 1997–2000. Pediatrics 111: 1159–1162.
121. Blondel B, Kaminski M (2002) Trends in the occurrence, determinants, and consequences of multiple births. Semin Perinatol 26: 239–249. doi:10.1053/sper.2002.34775.
122. Sellbom M, Ben-Porath YS (2005) Mapping the MMPI-2 Restructured Clinical scales onto normal personality traits: evidence of construct validity. J Pers Assess 85: 179–187. doi:10.1207/s15327752jpa8502_10.
123. Glicksohn J, Barrett TR (2003) Absorption and hallucinatory experience. Appl Cogn Psychol 17: 833–849. doi:10.1002/acp.913.
124. Harkness AR, McNulty JL, Ben-Porath YS (1995) The Personality Psychopathology Five (PSY-5): Constructs and MMPI-2 scales. Psychol Assess 7: 104–114. doi:10.1037/1040-3590.7.1.104.
125. Berenbaum SA, Taylor MA, Cloninger CR (1994) Family study of schizophrenia and personality. J Abnorm Psychol 103: 475–484. doi:10.1037/0021-843X.103.3.475.
126. Krueger RF, Hicks BM, Patrick CJ, Carlson SR, Iacono WG, et al. (2002) Etiologic connections among substance dependence, antisocial behavior and personality: Modeling the externalizing spectrum. J Abnorm Psychol 111: 411–424. doi:10.1037/0021-843X.111.3.411.
127. Blanchard JJ, Mueser KT, Bellack AS (1998) Anhedonia, positive and negative affect, and social functioning in schizophrenia. Schizophr Bull 24: 413–424. doi:10.1093/oxfordjournals.schbul.a033336.
128. Trzaskowski M, Yang J, Visscher PM, Plomin R (2013) DNA evidence for strong genetic stability and increasing heritability of intelligence from age 7 to 12. Mol Psychiatry. Available: http://www.nature.com/doifinder/10.1038/mp.2012.191. Accessed 15 July 2013.
129. Briley DA, Tucker-Drob EM (2013) Explaining the increasing heritability of cognitive ability across development: a meta-analysis of longitudinal twin and adoption studies. Psychol Sci. Available: http://pss.sagepub.com/lookup/doi/10.1177/0956797613478618. Accessed 15 July 2013.
130. Berg V, Rotkirch A, Väisänen H, Jokela M (2013) Personality is differentially associated with planned and non-planned pregnancies. J Res Pers 47: 296–305. doi:10.1016/j.jrp.2013.01.010.
131. Jokela M (2012) Birth-cohort effects in the association between personality and fertility. Psychol Sci 23: 835–841. doi:10.1177/0956797612439067.
132. Sartorius GA, Nieschlag E (2010) Paternal age and reproduction. Hum Reprod Update 16: 65–79. doi:10.1093/humupd/dmp027.
133. Gapminder Foundation (2012) Gapminder World. Available: http://www.gapminder.org/.
134. McMurry M (1993) Fertility rates rise for older women in 1980s. Popul Notes: 1–6.
135. Posthuma D, De Geus EJ, Bleichrodt N, Boomsma DI (2000) Twin-singleton differences in intelligence? Twin Res Hum Genet 3: 83–87. doi:10.1375/twin.3.2.83.
136. Derom R, Orlebeke J, Eriksson A, Thiery M (1995) The epidemiology of multiple births in Europe. In: Keith LG, Papiernik E, Keith DM, Luke B, editors. Multiple Pregnancy: Epidemiology, Gestation, and Perinatal Outcome. New York: Parthenon Publishing Group. 145–162.
137. Fergusson DM, Woodward LJ (1999) Maternal age and educational and psychosocial outcomes in early adulthood. J Child Psychol Psychiatry 40: 479–489. doi:10.1111/1469-7610.00464.
138. Fergusson DMM, Lynskey MTT (1993) Maternal age and cognitive and behavioural outcomes in middle childhood. Paediatr Perinat Epidemiol 7: 77–91. doi:10.1111/j.1365-3016.1993.tb00604.x.
139. Wichman AL, Rodgers JL, MacCallum RC (2006) A multilevel approach to the relationship between birth order and intelligence. Pers Soc Psychol Bull 32: 117–127. doi:10.1177/0146167205279581.
140. Frisell T, Öberg S, Kuja-Halkola R, Sjölander A (2012) Sibling comparison designs: Bias from non-shared confounders and measurement error. Epidemiology 23: 713–720. doi:10.1097/EDE.0b013e31825fa230.
141. Billari FC, Goisis A, Liefbroer AC, Settersten RA, Aassve A, et al. (2011) Social age deadlines for the childbearing of women and men. Hum Reprod 26: 616–622. doi:10.1093/humrep/deq360.
142. The 1000 Genomes Project Consortium (2012) An integrated map of genetic variation from 1,092 human genomes. Nature 491: 56–65. doi:10.1038/nature11632.
143. Flynn JR (1984) The mean IQ of Americans: Massive gains 1932 to 1978. Psychol Bull 95: 29–51. doi:10.1037/0033-2909.95.1.29.
144. Flynn JR (1987) Massive IQ gains in 14 nations: What IQ tests really measure. Psychol Bull 101: 171–191. doi:10.1037/0033-2909.101.2.171.
145. Mingroni MA (2007) Resolving the IQ paradox: Heterosis as a cause of the Flynn effect and other trends. Psychol Rev 114: 806–829. doi:10.1037/0033-295X.114.3.806.
146. Sundet JM, Eriksen W, Borren I, Tambs K (2010) The Flynn effect in sibships: Investigating the role of age differences between siblings. Intelligence 38: 38–44. doi:10.1016/j.intell.2009.11.005.
147. Johnson W, Penke L, Spinath FM (2011) Heritability in the era of molecular genetics: Some thoughts for understanding genetic influences on behavioural traits. Eur J Pers 25: 254–266. doi:10.1002/per.836.
148. Wicherts JM, Dolan CV, Hessen DJ, Oosterveld P, van Baal GCM, et al. (2004) Are intelligence tests measurement invariant over time? Investigating the nature of the Flynn effect. Intelligence 32: 509–537. doi:10.1016/j.intell.2004.07.002.
149. Rodgers JL (1998) A critique of the Flynn Effect: massive IQ gains, methodological artifacts, or both? Intelligence 26: 337–356. doi:10.1016/S0160-2896(99)00004-5.
150. Teasdale TW, Owen DR (2000) Forty-year secular trends in cognitive abilties. Intelligence 28: 115–120. doi:10.1016/S0160-2896(99)00034-3.
151. Sundet JM, Barlaug DG, Torjussen TM (2004) The end of the Flynn effect? A study of secular trends in mean intelligence test scores of Norwegian conscripts during half a century. Intelligence 32: 349–362. doi:10.1016/j.intell.2004.06.004.
152. Teasdale TW, Owen DR (2005) A long-term rise and recent decline in intelligence test performance: The Flynn Effect in reverse. Pers Individ Differ 39: 837–843. doi:10.1016/j.paid.2005.01.029.
153. Teasdale TW, Owen DR (2008) Secular declines in cognitive test scores: A reversal of the Flynn Effect. Intelligence 36: 121–126. doi:10.1016/j.intell.2007.01.007.
154. Golding J, Steer C, Pembrey M (2010) Parental and grandparental ages in the autistic spectrum disorders: a birth cohort study. PLoS ONE 5: e9939. doi:10.1371/journal.pone.0009939.
